# Robustness and fault tolerance make brains harder to study

**DOI:** 10.1186/1741-7007-9-46

**Published:** 2011-06-29

**Authors:** Shyam Srinivasan, Charles F Stevens

**Affiliations:** 1 2200 Gross Hall, University of California, Irvine, CA 92697, USA; 2 The Salk Institute, 10010 North Torrey Pines Road, La Jolla, CA 92037, USA

## Abstract

Brains increase the survival value of organisms by being robust and fault tolerant. That is, brain circuits continue to operate as the organism needs, even when the circuit properties are significantly perturbed. Kispersky and colleagues, in a recent paper in *Neural Systems & Circuits*, have found that Granger Causality analysis, an important method used to infer circuit connections from the behavior of neurons within the circuit, is defeated by the mechanisms that give rise to this robustness and fault tolerance.

**See research article: **http://www.neuralsystemsandcircuits.com/content/1/1/9/abstract

## 

Neuroscience has always been concerned with how neural circuits in the brain manage to carry out the computations that underlie behavior, but there has been a recent resurgence of interest in circuits. This intensified interest largely reflects the development of impressive new techniques for discovering how the brain is wired [1], for perturbing interactions between individual neurons or neuron classes [2], and for observing the activity exhibited by many members of neuronal populations as they go about their job [3-5]. A new paper by Kispersky *et al*. [6], however, highlights the observation that brains are designed to make life hard for neuroscientists who want to understand how circuits work. Specifically, this group has shown that an important analytical technique - Granger Causality - can invent causal interactions that do not actually exist between neurons, and they have figured out why the method sometimes fails.

Clive Granger, a Nobel prize winning economist, developed a method designed to detect causal relations in econometric data, and the method has also been applied in neurobiology. To see how this method works, suppose neuron A has a direct synaptic input onto neuron B that helps to drive the output of B; the output of cell A (one of the many inputs into cell B) is designated by *a*(*t*), and the output of cell B by *b*(*t*), where *t *represents time. Generally, neuronal outputs reflect non-random inputs, so the output of cell B will have some temporal structure, which means that earlier parts of *b*(*t*) will be correlated with the later parts of *b*(*t*). To apply Granger Causality, it is first necessary to determine how well the earlier parts of *b*(*t*) will predict the later parts of *b*(*t*) because these correlations reflect the overall temporal structure in the total signal cell B receives, together with the properties of cell B's spike-encoding mechanisms. The next step is to determine whether a better prediction of *b*(*t*) at later times can be obtained by including information from earlier parts of *a*(*t*) as well as the earlier parts of *b*(*t*) itself. You then do a statistical test to find out if the improvement in predicting *b*(*t*) by including information from *a*(*t*) is a lot better than chance, and if it is, then you say that *a*(*t*) is causally related to *b*(*t*). In biological terms, this sort of causation would be produced by a synaptic input A → B, and so significant Granger Causality can be a sign of a direct synaptic connection between a pair of cells. It gives a way of inferring the brain's wiring diagram.

This argument seems fine and has been shown to work in earlier studies. Or perhaps it only seemed to work before because the test was not sufficiently rigorous. What Kispersky *et al*. found, however, is that Granger Causality analysis claimed a significant connection between a pair of cells in the crab pyloric network (a simple, extensively studied pattern generation network), even though this cell pair is known not to communicate directly. If Granger Causality analysis works in general, it certainly should work in this very simple, very well studied circuit, but it did not.

What went wrong? By carrying out modeling studies, where you can experiment with different inputs and circuit designs, Kispersky *et al*. examined a range of different cases: correlated noise, and a number of model three-neuron networks with various connection patterns. For all of these cases, Granger Causality analysis works just as expected and finds causal connections where they exist and not where they are absent. The final test was an analysis of a model for the pyloric ganglion (Figure 1a). The job of the pyloric network is to drive some mechanical filtering structures rhythmically so that small food particles are passed on to the hind gut for digestion while larger particles are kept back to be chewed up more in a structure called the gastric mill. The crab needs this network to be robust and fault tolerant: it has to work properly, even when parts of the network are perturbed quite a bit [7]. When the pyloric ganglion model used neurons that fired in an unnatural fashion, with the neurons firing in a variable arrhythmic pattern, Granger Causality analysis correctly predicted how the cells were connected (Figure 1b). However, when the pyloric ganglion model was generating its natural, rhythmic output, Granger Causality analysis failed for the model pyloric ganglion network just as it did for the behavior of the actual ganglion (Figure 1c). Granger Causality was claiming a causal connection that, though consistent with the pattern being generated, was not actually present. The missing connection was not needed and the pattern could be generated by other connections that were present.

**Figure 1:**
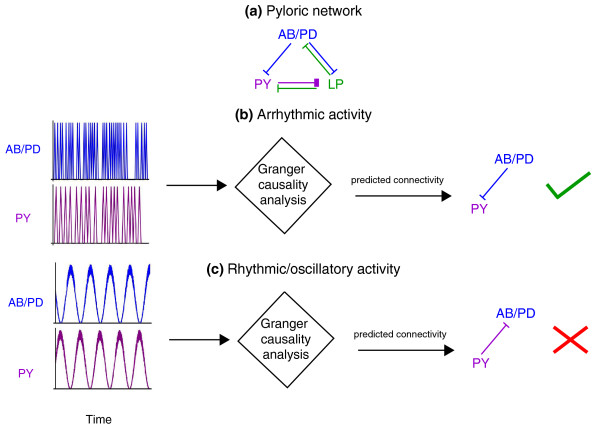
**The utility of Granger Causality analysis (GCA) as a tool for gauging connectivity is dependent on the nature of neural activity**. **(a) **Schematic of the pyloric network. Links denote inhibitory synapses, with color signifying direction. **(b) **Activity (schematic) of AB/PD and PY neurons in a model of this network where the activity of AB/PD and PY neurons is arrhythmic or non-oscillatory. When GCA was used, it correctly predicted a functional connectivity from PD to PY that matched known synaptic connectivity. **(c) **Activity (schematic) of AB/PD and PY neurons in a model where the activity of AB/PD and PY neurons is rhythmic or oscillatory, as in the naturally functioning circuit. Notably, although PD is not causally related to PY, activity of PD neurons followed activity of PY neurons. When GCA was used, it predicted a functional connectivity from PY to PD that did not match known synaptic connectivity. AB/PD, anterior burster/pyloric dilator; LP, lateral pyloric; PY, pyloric.

Invertebrates have many pattern generation networks. This same problem would be expected to arise in any of them because they all have been designed to keep working even when something goes wrong with the network. One might ask whether this is a problem unique to invertebrates who have very simple (numerically, at least) neural circuits. Actually, the problem is likely to be worse in the vertebrate brain because vertebrates rely on redundant neurons in their circuits to achieve fault tolerance. The logic behind the use of redundant neurons to produce fault tolerance is that the overall pattern generated does not depend on any single connection being present. No two of these networks have exactly the same connections, but they still work as they need to, and they continue working even when connections or cells are eliminated (up to a point).

A very nice analysis of this phenomenon in mammals has been carried out by Schwab *et al*. [8] for the preBotziner network, a pattern generator for breathing. Because of redundancies in this network, its output is invariant as individual neurons are removed (up to a critical number) and in such a network, analytical techniques (such as Granger Causality) would be expected to identify synaptic connections between neurons even where none exist. Although this example is for a pattern generator, the same principle of fault tolerance through redundancy holds for all sorts of networks, and they all present the same problem for the application of Granger Causality.

In summary, neural networks have been designed to have outputs that degrade gracefully as network elements are eliminated or their properties perturbed. Such a design principle makes the networks work better for the animals, but simultaneously makes life harder for neuroscientists who want to learn how the network works by making measurements on the network as it does its job.

## References

[B1] Luo L Callaway EM Svoboda K Genetic dissection of neural circuits Neuron 2008 57 634 660 10.1016/j.neuron.2008.01.002 18341986 PMC2628815

[B2] Airan RD Thompson KR Fenno LE Bernstein H Deisseroth K Temporally precise *in vivo *control of intracellular signalling Nature 2009 458 1025 1029 10.1038/nature07926 19295515

[B3] Ohki K Chung S Ch'ng YH Kara P Reid RC Functional imaging with cellular resolution reveals precise micro-architecture in visual cortex Nature 2005 433 597 603 10.1038/nature03274 15660108

[B4] Bock DD Lee WC Kerlin AM Andermann ML Hood G Wetzel AW Yurgenson S Soucy ER Kim HS Reid RC Network anatomy and *in vivo *physiology of visual cortical neurons Nature 2011 471 177 182 10.1038/nature09802 21390124 PMC3095821

[B5] Briggman KL Helmstaedter M Denk W Wiring specificity in the direction-selectivity circuit of the retina Nature 2011 471 183 188 10.1038/nature09818 21390125

[B6] Kispersky T Gutierrez GJ Marder E Functional connectivity in a rhythmic inhibitory circuit using Granger causality Neural Systems Circuits 2011 1 9 10.1186/2042-1001-1-9 PMC3314404 22330428

[B7] Prinz AA Bucher D Marder E Similar network activity from disparate circuit parameters Nat Neurosci 2004 7 1345 1352 10.1038/nn1352 15558066

[B8] Schwab DJ Bruinsma RF Feldman JL Levine AJ Rhythmogenic neuronal networks, emergent leaders and k-cores Phys Rev E Stat Nonlin Soft Matter Phys 2010 82 051911 21230504 21230504 10.1103/PhysRevE.82.051911 PMC3477876

